# The quantitative enamel firing technique based on regression analysis

**DOI:** 10.1371/journal.pone.0322459

**Published:** 2025-05-19

**Authors:** Yaqin Qian, Xiangdong Dai

**Affiliations:** 1 Hunan First Normal University, Changsha, China; 2 Central South University of Forestry and Technology, Changsha, China; Hamadan University of Medical Sciences, IRAN, ISLAMIC REPUBLIC OF

## Abstract

Enamel firing, as a key technique of enamel craftsmanship, has had no quantitative standards to follow since ancient times. To solve this problem, linear regression analysis was used to conduct quantitative enamel firing experiments. Through enamel firing experiments on enamel specimens with different masses, the linear regression equation was solved using the obtained experimental data and the regression equation was subjected to the analysis of variance. The experimental results indicate that the mass of enamel specimens has a real linear regression relationship with the firing duration. To verify the applicability of the linear regression equation, the equation was applied and validated. That is, enamel specimens with different specifications and colors were prepared, the firing durations of which were then calculated using the regression equation. The enamel specimens were fired according to the calculated firing durations, all obtaining optimal enamel decorations. The following conclusions are drawn: simulating enamel firing using the linear regression equation established in the quantitative enamel firing experiments can realize a quantitative, standardized enamel firing technique.

## Introduction

Enamels refer to firing the mixture of quartz, feldspar, saltpeter, and sodium carbonate with related metallic oxides and then smashing the resultant glassy frits (enamel glazes), which are applied onto metal surfaces and fired to form enamel surfaces in different colors [1]. Enamels are beautiful decorative materials, and decorations or wares made of enamels are called enamel products. Enamel products show the advantages not only of glass, but also of metals, while are not fragile and will not rust (*c.f*. glass or metal). They are characterized by rust resistance, smoothness, cleanliness, aesthetics, wear resistance, corrosion resistance, and high chemical stability [2]. Since the Yuan dynasty (AD 1206–1368) when enamels were introduced to China, enamel craftsmanship had made a great progress in the middle Ming dynasty (1368–1644), when a filigree enamel brand famous in China and abroad was founded [3,4]. To the times of Qianlong emperor in the Qing dynasty (AD 1644–1912), the art of enamels had reached its peak. Enamel-decorated furniture had become a major characteristic of palace furniture of the Qing dynasty, which is resplendent, magnificent, and colorful, highlighting the elegant and grand temperament. It belongs to a national treasure and precious cultural relic and a quintessence of Chinese art [5–9]. On May 20, 2006, the craftsmanship of Cloisonné has been approved by the State Council of China to be listed in the first batch of national intangible cultural heritage.

If divided according to the craftsmanship, enamels can be categorized into several types, including filigree, basse-taille, painted, and transparent enamels. The enamel craftsmanship is exquisite and complex; this is particularly the case for filigree enamel craftsmanship (dubbed Cloisonné), involving eight basic technological processes, namely: enamel glaze making, copper pad preparation, wire inlay, soldering, enamel-filling, enamel firing, polishing, and gold-plating. A total of 109 technological processes at different levels are involved [10]. In the manufacturing process, satisfactory enamel products cannot be obtained unless being meticulously and repeatedly processed by craftsmen. When using either technique to make enamels, firing is always a necessary step. The process refers to placing copper pads coated with enamel glazes into a high-temperature kiln to be fired at high temperatures ranging from 800 to 850 °C [10–12]. Firing and polishing are needed each time after applying enamel glazes. The most striking aspect of Cloisonné is enamel firing-induced and enamels become more beautiful every time after enamel firing, which is described by the peer as “the phoenix rises from the ashes”. However, because the firing is performed rapidly at high temperatures, craftsmen find difficulty in accurately deciding upon the firing duration; instead, they mainly depend on their experience. They need to judge the firing duration in accordance with the heating duration and degree [11]. If the firing duration is too short, enamel glazes cannot be completely melted and therefore cannot bind as tightly to the copper pads. As a result, the enamels are not only not beautiful but also readily crack and burst apart. If the firing duration is too long, enamel glazes flow away from copper pads, so enamel glazes need to be reapplied. Hence, such an enamel firing method completely reliant on experience can hardly guarantee the consistency of product quality from batch to batch, especially when firing small enamel decorations, which are frequently overfired or underfired. Therefore, how to achieve quantitative enamel firing, that is, calculating firing duration and controlling enamel quality according to factors including kiln temperature and mass of enamel decorations, becomes a technological problem that needs to be solved. After reviewing both the Chinese and foreign literature, no research on the quantitative enamel firing has been reported. Therefore, quantitative research into enamel firing techniques represents an innovative exploration of the traditional enamel technology. This not only can realize a quantitative, standardized enamel firing technique but also can improve the control of enamel quality and repeatability, thus achieving the innovation and inheritance of enamel firing, an intangible heritage technology.

## 1 Materials and methods

### 1.1 Materials

#### 1.1.1 Experimental materials.

The experimental materials included red copper plates (pure copper plates) with the thickness of 2 and 4 mm, high-temperature enamel glazes in four colors (porcelain-white, silver-coral-red, copper-yellow, and sky-blue ones), and (self-made) 3% sticky rice glue.

#### 1.1.2 Instruments and equipment.

**Fig 1:**
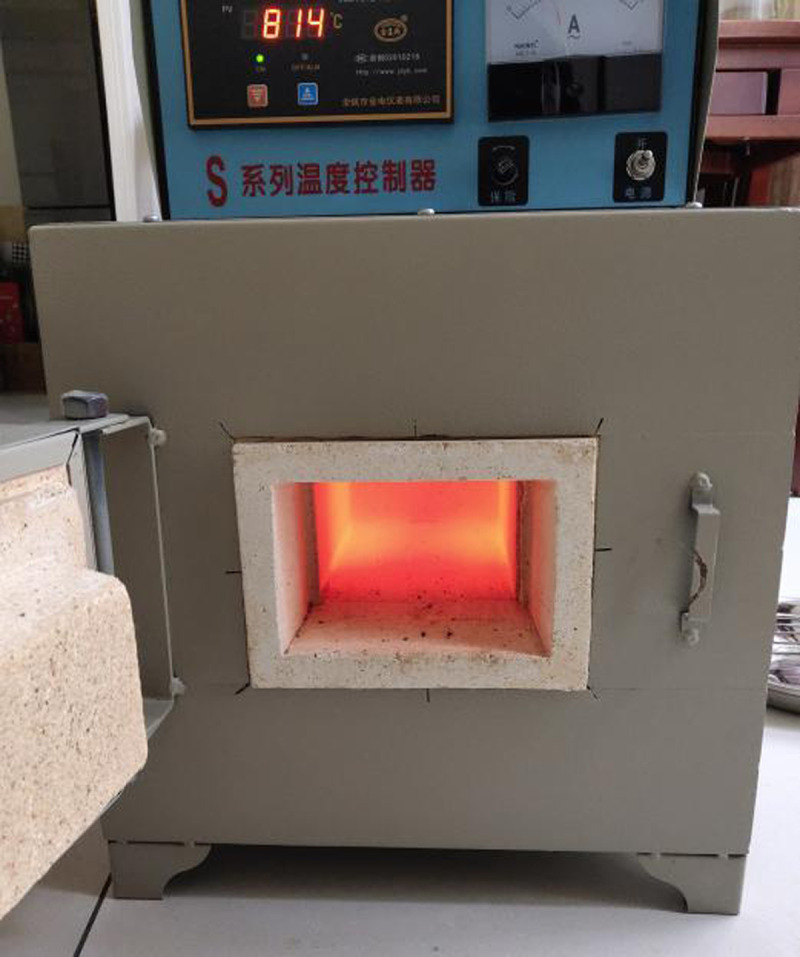
Electric muffle stove.

Instruments and equipment included: ① an electric muffle stove (Fig 1) produced by Shangyu Daoxu Kexi instrument factory in Shaoxing City (Zhejiang Province, China), with a power supply of 220 V at 13 A, a rated power of 2.5 kW, furnace volume of 200 mm × 120 mm × 80 mm, and maximum rated temperature of 1200 °C; ② a Wante precision electronic balance produced by Hangzhou Wante Weighing Instrument Co., Ltd with a weighing precision of 0.01 g. The electronic balance used a power supply (220 V, 50 Hz) and had a maximum rated power consumption of 3 W.

### 1.2 Methods

#### 1.2.1 Design of experimental treatments.

The experiments aimed to ascertain the firing duration of enamel decorations and discern whether the firing duration is related to the mass of enamel decorations or not. Considering this, the influence of a single factor, namely, the mass of enamel decorations (enamel specimens) on the firing duration was only considered in the experiments. The factor of the specimen mass was designed at six treatment levels, which implied that the experiments involved one factor at six levels. The mass of specimen 1 (*X*_1_) was set to 37.6 g (mass of copper pads + mass of enamel glazes), which was taken as the benchmark mass. The mass of specimens in subsequent treatments successively increased by one benchmark mass. For example, the mass of specimen 2 (*X*_2_) was two benchmark masses (75.2 g); likewise, the mass of specimen 6 (*X*_6_) is six benchmark masses (225.6 g). The design of each experimental treatment level is summarized in Table 1.

**Table 1:** Experimental treatment levels.

Specimen	Specimen 1 (*X*_1_)	Specimen 2 (*X*_2_)	Specimen 3 (*X*_3_)	Specimen 4 (*X*_4_)	Specimen 5 (*X*_5_)	Specimen 6 (*X*_6_)
Volume of copper pads	50 × 40 × 2 mm = 4000 mm^3^	80 × 50 × 2 mm = 8000 mm^3^	120 × 50 × 2 mm = 12,000 mm^3^	100 × 80 × 2 mm = 16,000 mm^3^	10 0 × 100 × 2 mm = 20,000 mm^3^	120 × 100 × 2 mm = 24,000 mm^3^
Mass of copper pads	35.6 g	35.6 g × 2 = 71.2 g	35.6 g × 3 = 106.8 g	35.6 g × 4 = 142.4 g	35.6 g × 5 = 178 g	35.6 g × 6 = 213.6 g
Mass of enamel glazes	2 g	2 g × 2 = 4 g	2 g × 3 = 6 g	2 g × 4 = 8 g	2 g × 5 = 10 g	2 g × 6 = 12 g
Specimen mass	37.6 g	37.6 g × 2 = 75.2 g	37.6 g × 3 = 112.8 g	37.6 g × 4 = 150.4 g	37.6 g × 5 = 188 g	37.6 g × 6 = 225.6 g

Note: Copper pads were laser-cut and therefore had standard dimensions; through tests and calibration, the density of red copper was 8.9 g/cm^3^ in the calculation; the specimen mass = mass of copper pads + mass of enamel glazes.

#### 1.2.2 Experimental procedures.

To reduce the experimental error, other influencing factors in each treatment were all designed same in the experiments. That is, a same electric muffle stove was adopted to fire enamels and the firing temperature in each time was set to 820 °C. The operating enamel firing instructions below were strictly followed:

① The electric muffle stove has to be preheated before enamel firing: the power switch is turned on and the stove temperature is set to 500 °C to preheat the stove for 1 h. Then, the furnace temperature is adjusted to 820 °C to preheat the stove for 2 h. The preheating is conducted to evaporate moisture in the electric muffle stove, increase the temperature therein, and weaken temperature fluctuations during enamel firing.
② During enamel firing, it should be prepared in advance when placing the specimens (decorations) in and taking them out of the stove. The duration of opening the stove door should be controlled within 5 s in each time.
③ The stove temperature should be increased to 820 °C before placing specimens (decorations) in the stove in each time.
④ Calculating the firing duration: it starts from opening the stove door. Once it reaches the firing duration, the door is opened immediately to take the specimens (decorations) out.

To observe the enamel firing effect and reduce the experimental error, single-color (porcelain-white) enamel specimens were used to conduct enamel firing experiments.

#### 1.2.3 Statistical method of experimental results.

The linear regression analysis was used for statistical analysis of the quantitative enamel firing experimental results. That is, enamel specimens with different masses were designed and fired, of which the ideal firing durations were separately tested to evaluate the relationship between the mass of enamel specimens and the firing duration. The mass *X* of enamel specimens was taken as an independent variable while the firing duration *Y* needed to prepare ideal enamel specimens was taken as the dependent variable. The values of regression intercept *a* and regression coefficient *b* were calculated using the least squares method based on the general equation $\hat{y}\;$= *a* + *b*x for linear regression analysis. Then, the linear regression equation of the mass and firing duration of enamel specimens was solved, and the resulting equation was subjected to analysis of variance, to assess the significance of the regression relationship. In quantifying the significance of the linear regression equation, *F*-tests were adopted in the hypothesis test. If the regression relationship is significant, that is, *F* > *F*_0.05_ or *F* ≥ *F*_0.01_, it means that the mass of enamel specimens has a real linear regression relationship with the firing duration [13,14]. The research result provides a scientific basis for quantifying and standardizing the enamel firing technique.

#### 1.2.4 Specimen preparation.

**Fig 2:**
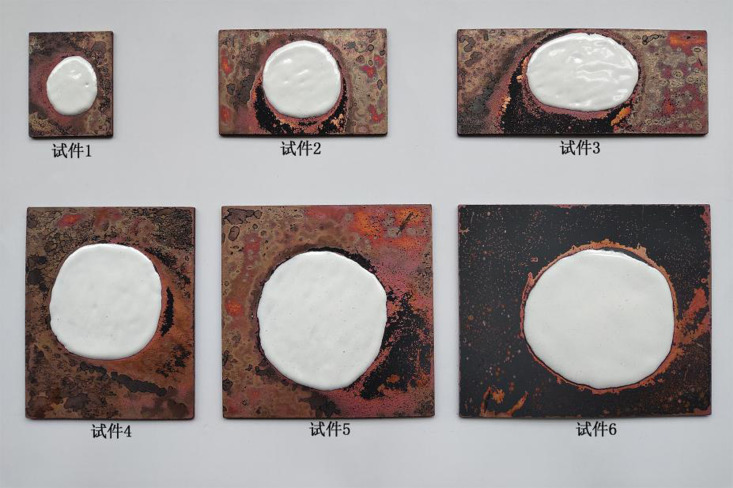
Enamel specimens with the porcelain-white enamel glaze.

Table 1 lists six types of porcelain-white enamel specimens designed with different masses, which were used to estimate the effect of firing duration. Specimen 1 had the lowest mass, so was taken as the benchmark for other enamel firing specimens, which meant that enamel firing experiments started from specimen 1. Due to the lack of firing durations for reference, many firing durations were set for the specimens, and also many such specimens were prepared for enamel firing experiments. Considering this, the research planned to make 15 specimens 1. Starting with specimens 2, due to the availability of the ideal firing duration of specimens 1 for reference, specimens 2 needed to be tested fewer times to determine their firing duration. Therefore, only five pieces were prepared separately for specimens 2, 3, 4, 5, and 6. That is, a total of 40 specimens were prepared for the six types of specimens using the following steps:

① **Making copper pads.** Copper pads were made of red copper plates (pure copper plates) with a thickness of 2 mm. A factory was entrusted to manufacture copper pads by laser cutting. Small copper plates, namely, copper pads of specimens, with six different specifications were cut following the design requirements in Table 1. Copper pads of specimens 1, 2, 3, 4, 5, and 6 separately measured 50 × 40 mm, 50 × 80 mm, 50 × 120 mm, 100 × 80 mm, 100 × 100 mm, and 100 × 120 mm.
② **Applying enamel glazes.** Applying enamel glazes refers to putting porcelain-white enamel glaze on copper pads. In the process, the masses of enamel glazes for specimens 1 ~ 6 should be weighed strictly following the designed masses of enamel glazes on each specimen. The weighed enamel glaze was placed at the center of the upper surface of each small copper plate, then wetted and mixed with self-made 3% sticky rice glue, and flattened to a 2-mm thick enamel glaze layer (Fig 2). After being naturally dried, the specimens were deemed to have been successfully prepared.

#### 1.2.5 Enamel firing experiments.

**Fig 3:**
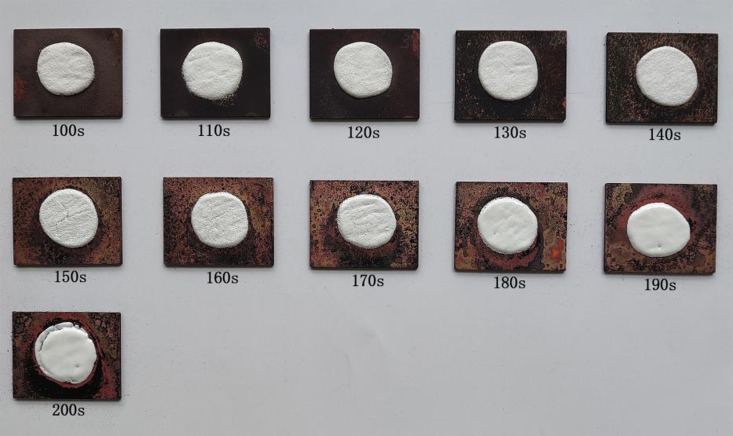
Enamel decorations of specimens 1 fired for different durations.

**Fig 4:**
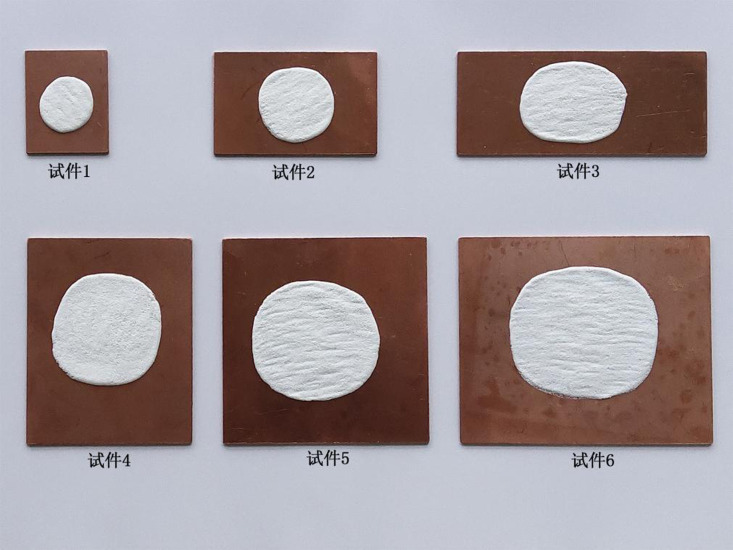
Optimal enamel decorations of specimens with the six specifications.

Specimens 1 have the lowest mass, so they absorb the least heat in the high-temperature stove (electric muffle stove) during enamel firing. That is to say, specimens 1 take the shortest time to be fired at a given stove temperature. Considering this, enamel firing experiments were designed to start from specimens 1 to first evaluate the ideal firing duration of specimens 1, which was taken as the benchmark firing duration of other specimens. The enamel firing steps are described as follows:

**Step 1: Assessing the ideal firing duration of specimens 1.** The firing duration of specimens 1 began from 100 s and subsequent durations were all 10 s longer than the previous ones. The firing duration did not exceed 200 s, when the enamel glaze began to flow, implying that the firing duration was too long. A total of 11 firing durations were evaluated. Enamel decorations prepared by firing specimens 1 for different durations are illustrated in Fig 3. Enamel decorations obtained by firing for different durations were observed and those with the optimal quality were selected (Fig 4). The firing duration of enamel decorations with the optimal quality is the ideal firing duration of specimens 1. Such enamel decorations show bright enamel surfaces with no fine particles and flows. If there are fine particles on enamel surfaces, it means that the enamel glaze is not completely molten, that is, the firing duration is so short that the heating duration and degree are insufficient (underfired). If the enamel glaze flows, this indicates that the enamel glaze has been completely molten and begins to flow, that is, the firing duration is excessive (overfired). Results pertaining to specimens 1 after different firing durations are listed in Table 2.

**Table 2:** Test results pertaining to specimens 1 after different firing durations.

Firing duration	Enamel firing effect
100 s	The enamel glazes are not molten, which means that the firing duration is far from enough.
110 s	The enamel glaze is not molten, which means that the firing duration is far from enough.
120 s	The enamel glaze is not molten, which means that the firing duration is far from enough.
130 s	Some enamel glazes begin to be molten, which means that the firing duration is far from enough.
140 s	Some enamel glazes begin to be molten, which means that the firing duration is far from enough.
150 s	Some enamel glazes begin to be molten, which means that the firing duration is far from enough.
160 s	Some enamel glazes begin to be molten, which means that the firing duration is far from enough.
170 s	Most enamel glaze has been molten, which means that the firing duration is obviously not long enough.
180 s	The vast majority of enamel glaze has been molten, which means that the firing duration is still not long enough.
190 s	The enamel glaze has been completely molten and the enamel shows ideal quality.
200 s	The enamel glaze has been completely molten and begins to flow, which indicates that the firing duration is too long.

**Step 2: Assessing the ideal firing duration of specimens 2.** The mass of specimens 2 is one benchmark mass (37.6 g) greater than that of specimens 1, so the ideal firing duration of specimens 2 is theoretically longer than that of specimens 1. Therefore, the firing durations of specimens 2 can be designed according to the ideal firing duration of specimens 1. The ideal firing duration of specimens 1 is taken as the initial (shortest) firing duration of specimens 2, and subsequent durations successively increase by 10 s. Totally five firing durations are designed. Enamel decorations fired for different durations are found to select the optimal one (Fig 4). The firing duration of enamel decoration with the optimal quality is the ideal firing duration of specimens 2. Test results pertaining to specimens 2 after different firing durations are listed in Table 3.

**Table 3:** Test results pertaining to specimens 2 after different firing durations.

Firing duration	Enamel firing effect
190 s	Most enamel glaze has been molten, which means that the firing duration is obviously not long enough.
200 s	The vast majority of enamel glaze has been molten, which means that the firing duration is still not long enough.
210 s	The enamel glaze has been completely molten and the enamel shows ideal quality.
220 s	The enamel glaze has been completely molten while flows, which means that the firing duration is too long.
230 s	The enamel glaze has been completely molten and begins to flow, which means that the firing duration is too long.

**Step 3: Evaluating the ideal firing duration of specimens 3.** Because the mass of specimens 3 is one benchmark mass (37.6 g) heavier than that of specimens 2, theoretically the ideal firing duration of specimens 3 is longer than that of specimens 2. So, the firing durations of specimens 3 can be set based on the ideal firing duration of specimens 2. The ideal firing duration of specimens 2 is taken as the initial (shortest) firing duration of specimens 3, and subsequent durations successively prolong by 10 s. A total of five firing durations are designed. Enamel decorations fired for different durations are found to select the optimal one (Fig 4). The firing duration of enamel decoration with the optimal quality is the ideal firing duration of specimens 3. Test results pertaining to specimens 3 after different firing durations are listed in Table 4.

**Table 4:** Test results pertaining to specimens 3 after different firing durations.

Firing duration	Enamel firing effect
210 s	A small amount of enamel glaze is molten, which means that the firing duration is far from enough.
220 s	Most enamel glaze has been molten, which means that the firing duration is obviously not long enough.
230 s	The vast majority of enamel glaze has been molten, which means that the firing duration is still not long enough.
240 s	The enamel glaze has been completely molten and the enamel shows ideal quality.
250 s	The enamel glaze has been completely molten while flowed, which means that the firing duration is too long.

**Step 4: Assessing the ideal firing duration of specimens 4.** Considering that the mass of specimens 4 is one benchmark mass (37.6 g) heavier than that of specimens 3, the ideal firing duration of specimens 4 is theoretically longer than that of specimens 3. Hence, the firing durations of specimens 4 can be set in accordance with the ideal firing duration of specimens 3. The ideal firing duration of specimens 3 is used as the initial (shortest) firing duration of specimens 4, and subsequent durations successively increase by 10 s. A total of five firing durations are designed. Enamel decorations fired for different durations are found to select the optimal one (Fig 4). The firing duration of enamel decoration with the optimal quality is the ideal firing duration of specimens 4. Test results pertaining to specimens 4 after different firing durations are displayed in Table 5.

**Table 5:** Test results pertaining to specimens 4 after different firing durations.

Firing duration	Enamel firing effect
240 s	A small amount of enamel glaze is molten, which means that the firing duration is far from enough.
250 s	Some enamel glazes have been molten, which means that the firing duration is obviously not long enough.
260 s	Most enamel glaze has been molten, which means that the firing duration is obviously not long enough.
270 s	The vast majority of enamel glaze has been completely molten, which means that the firing duration is still not long enough.
280 s	The enamel glaze has been completely molten and the enamel shows ideal quality.

**Step 5: Assessing the ideal firing duration of specimens 5.** The mass of specimens 5 is one benchmark mass (37.6 g) greater than that of specimens 4, so the ideal firing duration of specimens 5 is theoretically longer than that of specimens 4. So, the firing durations of specimens 5 can be set in accordance with the ideal firing duration of specimens 4. The ideal firing duration of specimens 4 is taken as the initial (shortest) firing duration of specimens 5, and subsequent durations successively increase by 10 s. A total of five firing durations are designed. Enamel decorations fired for different durations are shown to select the optimal one (Fig 4). The firing duration of enamel decoration with the optimal quality is the ideal firing duration of specimens 5. Test results pertaining to specimens 5 after different firing durations are shown in Table 6.

**Table 6:** Test results pertaining to specimens 5 after different firing durations.

Firing duration	Enamel firing effect
280 s	Most enamel glaze has been molten, which means that the firing duration is obviously not long enough.
290 s	The vast majority of enamel glaze has been completely molten, which means that the firing duration is still not long enough.
300 s	The enamel glaze has been completely molten and the enamel shows ideal quality.
310 s	The enamel glaze has been completely molten while flows, which means that the firing duration is too long.
320 s	The enamel glaze has been completely molten and begins to flow, which means that the firing duration is too long.

**Step 6: Assessing the ideal firing duration of specimens 6.** As the mass of specimens 6 is one benchmark mass (37.6 g) greater than that of specimens 5, the ideal firing duration of specimens 6 is theoretically longer than that of specimens 5. Considering this, the firing durations of specimens 6 can be set in accordance with the ideal firing duration of specimens 5. The ideal firing duration of specimens 5 is set as the initial (shortest) firing duration of specimens 6, and subsequent durations successively increase by 10 s. A total of five firing durations are designed. Enamel decorations fired for different durations select the optimal one (Fig 4). The firing duration of enamel decoration with the optimal quality is the ideal firing duration of specimens 6. Table 7 lists the test results pertaining to specimens 6 after different firing durations.

**Table 7:** Test results pertaining to specimens 6 after different firing durations.

Firing duration	Enamel firing effect
300 s	Some enamel glazes have been molten, which means that the firing duration is obviously not long enough.
310 s	Most enamel glaze has been molten, which means that the firing duration is evidently not long enough.
320 s	The vast majority of enamel glaze has been completely molten, which means that the firing duration is still not long enough.
330 s	The enamel glaze has been completely molten and the enamel shows ideal quality.
340 s	The enamel glaze has been completely molten and has flows, which means that the firing duration is too long.

## 2 Results

### 2.1 Analysis of the results

#### 2.1.1 Solving the regression equation.

The ideal firing durations obtained by testing porcelain-white enamel specimens with different masses above are listed in Table 8.

**Table 8:** Ideal firing durations of porcelain-white enamel specimens with different masses.

Specimens	1	2	3	4	5	6
Specimen mass (*x*)	37.6 g	75.2 g	112.8 g	150.4 g	188 g	225.6 g
Ideal firing duration (*y*)	190 s	210 s	240 s	280 s	300 s	330 s

Then, it can be calculated that ∑x= 789.6, $\sum x^{2}=128652.16$, ∑y=1550, $\sum y^{2}=\;415100$, ∑xy=222968, $\overset{―}{x\;}=131.6$, and $\overset{―}{y}=258.33$; it is known that *n* = 6.

By using related calculation formulas, it is calculated that [13]:


$${SS}_{x}=\sum x^{2}\;–\;\frac{1}{n}\;(\sum x{)}^{2}=128652.16\;–\;\frac{1}{6}{\left(789.6\right)}^{2}=24740.8$$

$${SS}_{y}=\sum y^{2}\;–\;\frac{1}{n}\;(\sum y{)}^{2}=415100\;–\frac{1}{6}{\left(1550\right)}^{2}=14683.33$$

$$SP=\sum xy\;–\;\frac{1}{n}\;\left(\sum x\right)\left(\sum y\right)=222968\;–\frac{1}{6}(789.6\times 1550)=18988$$

The regression coefficient *b* is calculated as follows:


$$b=\frac{SP}{{SS}_{x}}=\frac{18988}{24740.8}=0.7675\;(s/g)$$

The regression intercept *a* is calculated as follows:


$$a=\overset{―}{y}–b\overset{―}{x}=258.33-0.7675\times 131.6=157.327(s)$$

The values of b and a are substituted into the general form of linear regression equation, thus obtaining the linear regression equation of experimental data in Table 8:


$$\hat{y}=a+bx=157.327+0.7675x$$

where x is the mass of enamel specimens (decorations); $\hat{y}$ denotes the firing duration and its value changes with x. That is, the firing duration changes with the mass of enamel specimens (decorations).

#### 2.1.2 Significance tests of the regression relationship.

*F*-tests are used for analysis of variance.

The number of degrees of freedom (DOF) and sum of squares are decomposed thus:

Discrete regression DOF is *v* = *n* – 2 = 6–2= 4

Regression DOF is *v* = (*n*−1) − (*n*−2) = 1

DOF of total variation is *v* = *n* – 1 = 6–1= 5

Discrete regression sum of squares is $Q={SS}_{y}–\;\frac{{\left(SP\right)}^{2}}{{SS}_{x}}=14683.33–\frac{18988^{2}}{24740.8}=\;110.4729$

Regression sum of squares is ${U=SS}_{y}–\;Q=\;14683.33–110.4729=\;14572.8571$

The sum of squares of total variation is calculated above as ${SS}_{y}=14683.33$

The *F*-value calculated using the formula is [13]:


$$F=\frac{\;\frac{{\left(SP\right)}^{2}}{{SS}_{x}}}{\frac{Q}{n-2}}=\frac{14572.8571}{27.6182}=527.65$$

Therefore, the obtained variance analysis results are listed in Table 9.

**Table 9:** Hypothesis test of regression relationship of data in Table 8.

Source of variation	DF	SS	MS	*F*	*F*_0.05_	*F*_0.01_
Regression	1	14572.8571	14572.8571	527.65	7.71	21.20
Discrete regression	4	110.4729	27.6182			
Total variation	5	14683.33				

The results in Table 9 show *F* = 527.65 in the hypothesis test result of the regression relationship. The *F*-value is not only greater than *F*_0.05_ (7.71) but also larger than *F*_0.01_ (21.20), indicating that the regression relationship is extremely significant and there is a real linear relationship between the mass and firing duration of enamel specimens. Moreover, the reliability of the linear regression relationship exceeds 99%. This means that the established linear regression equation $(\hat{y}\;$= 157.327 + 0.7675x) has statistical significance.

#### 2.1.3 Chart of the changing trend of the linear regression relationship.

**Fig 5:**
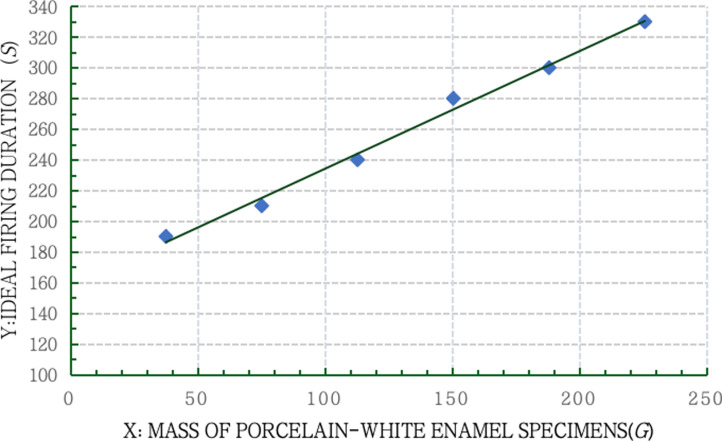
The relationship between the mass and firing duration of porcelain-white enamel specimens.

According to the linear regression equation attained above, data in Table 8 were plotted as a chart of the changing trend of the linear regression relationship (Fig 5).

As shown in Fig 5, the ideal firing duration of porcelain-white enamel specimens prolongs with the increasing mass of specimens in a linear manner.

### 2.2 Application of the results

The ideal firing durations of other enamel decorations were simulated using the linear regression equation ($\hat{y}\;$= 157.327 + 0.7675x) constructed based on the above experimental results, to verify whether the model is representative, scientific, and practical (or not) [15]. The applicability of the model can be validated from two aspects: one simulating the firing durations of larger porcelain-white enamel specimens (decorations), the other simulating the firing durations of colored (silver-coral-red, copper-yellow, and sky-blue) enamel specimens (decorations). Additionally, the simulated firing durations are used for enamel firing, to test the actual enamel firing effect.

#### 2.2.1 Calculating the firing durations of larger porcelain-white enamel specimens using the model.

Larger porcelain-white enamel specimens were designed to simulate their ideal firing durations using the linear regression equation ($\hat{y}\;$= 157.327 + 0.7675x), as listed in Table 10.

**Table 10:** Simulation of the ideal firing durations of porcelain-white enamel specimens.

Volume of copper pads of specimens	*X*: Specimen mass (mass of copper pads + mass of enamel glazes)	*Y*: Simulated ideal firing duration
100 mm × 80 mm × 4 mm = 32,000 mm^3^	284.8 g + 16 g = 300.8 g	388.19 s

**Fig 6:**
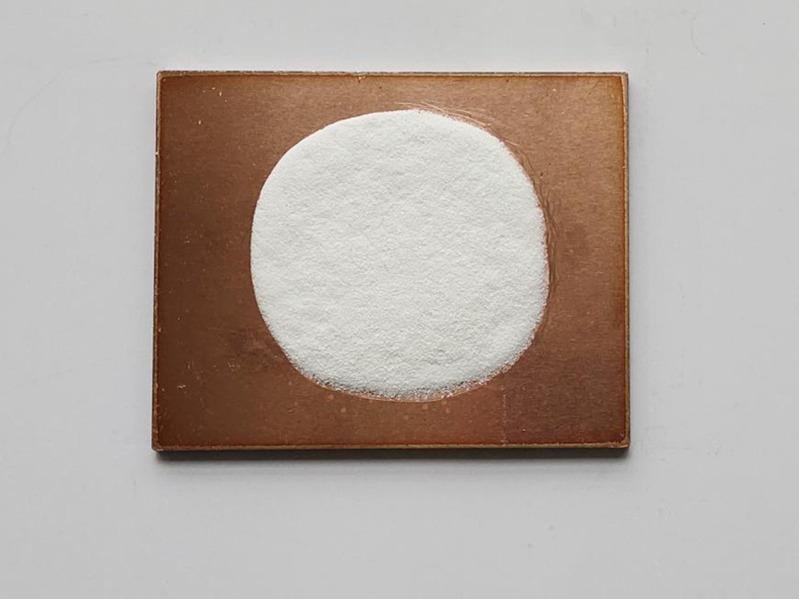
Larger porcelain-white enamel specimens.

**Fig 7:**
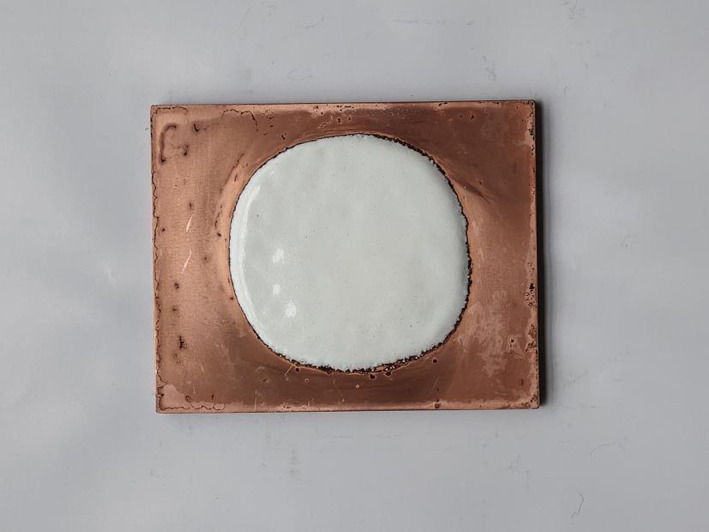
Enamel firing effect of larger porcelain-white enamel specimens.

Real objects of porcelain-white enamel specimens in Table 10 were prepared (Fig 6) and fired for the simulated ideal firing durations, with three duplicates. The actual enamel firing effect is shown in Fig 7.

As shown in Fig 7, enamel specimens in Fig 6 were fired according to the firing duration simulated using the linear regression equation for verification, and the fired porcelain-white enamel specimens showed the ideal effect.

#### 2.2.2 Calculating the firing durations of colored enamel specimens with different specifications using the model.

Colored (silver-coral-red, copper-yellow, and sky-blue) enamel specimens with two specifications were designed. Their ideal firing durations were simulated using the linear regression equation ($\hat{y}\;$= 157.327 + 0.7675x), as displayed in Table 11.

**Table 11:** Simulation of ideal firing durations of colored enamel specimens.

Specimen	Volume of copper pads of specimens	X: Specimen mass (mass of copper pads + mass of enamel glazes)	Y: Simulated ideal firing duration
Small specimen	50 mm × 40 mm × 2 mm = 4 cm^3^	35.6 g + 2 g = 37.6 g	186.19 s
Large specimen	100 mm × 80 mm × 4 mm = 32 cm^3^	284.8 g + 16 g = 300.8 g	388.19 s

**Fig 8:**
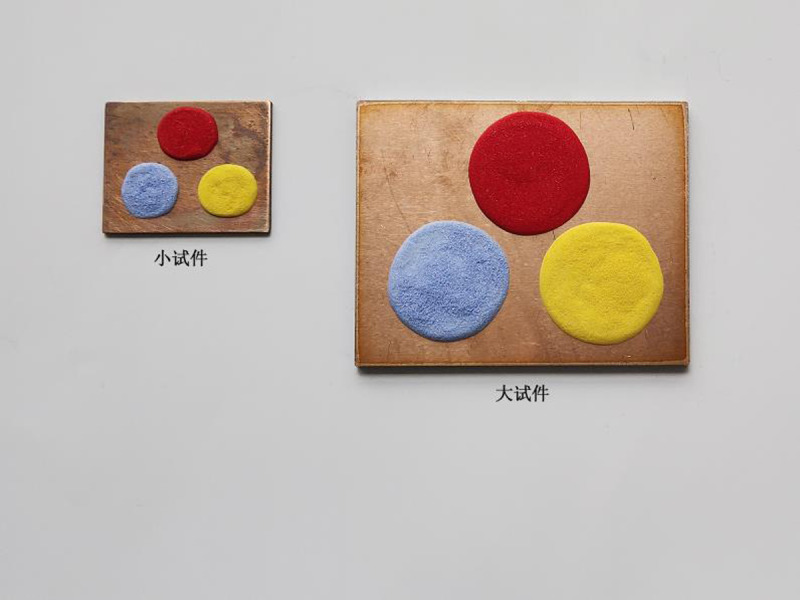
Colored enamel specimens with two specifications.

**Fig 9:**
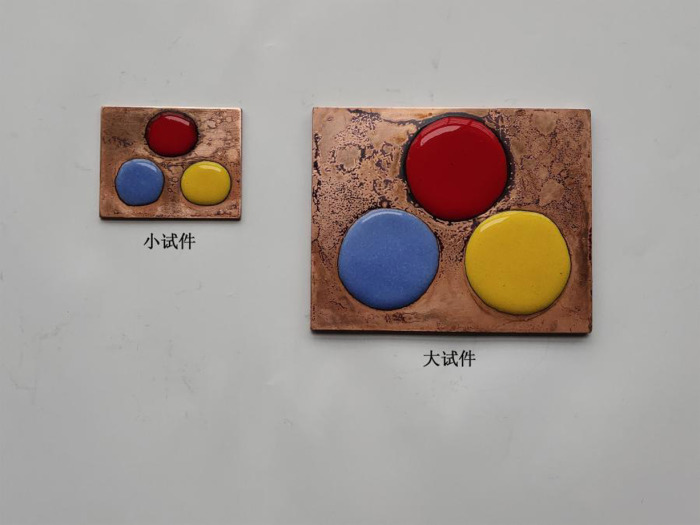
Enamel firing effect of colored enamel specimens with two specifications.

Real objects of colored enamel specimens with two specifications in Table 11 were prepared (Fig 8) and then fired for the simulated ideal firing durations, with three duplicates. The actual enamel firing effect is shown in Fig 9.

As shown in Fig 9, the colored enamel specimens in Fig 8 were fired according to the firing durations simulated using the linear regression equation for verification. The obtained colored enamel specimens all reach the ideal effect.

## 3 Discussion

**Fig 10:**
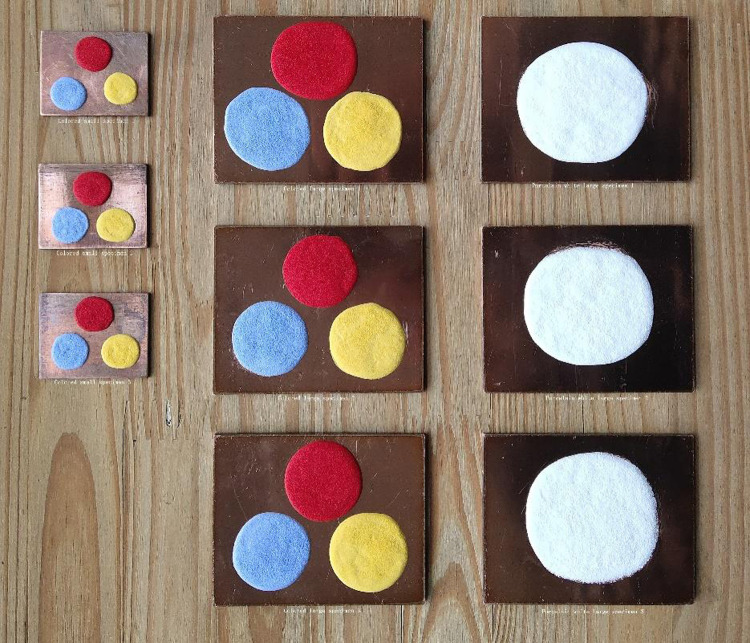
Three types of enamel specimens (three pieces for each).

**Fig 11:**
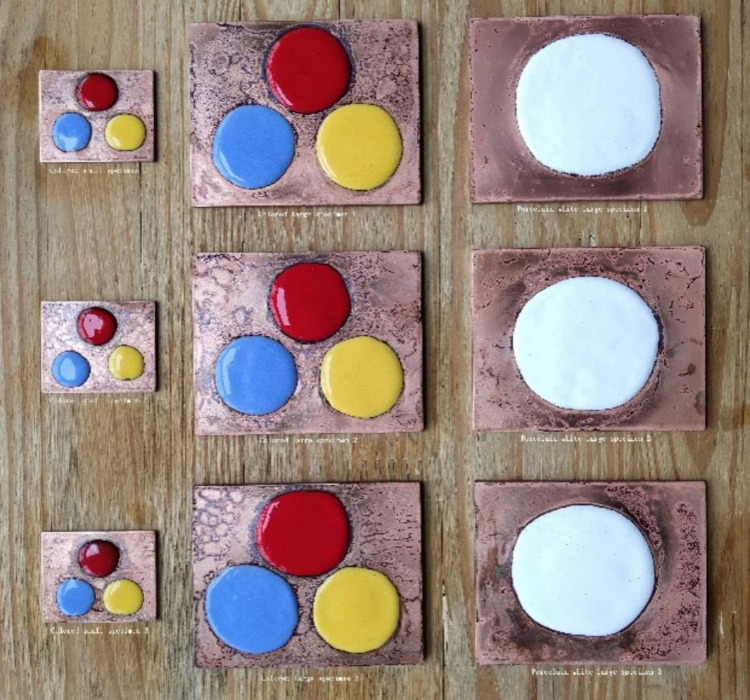
Enamel firing effects of three duplicates of the three types of enamel specimens.

Little quantitative research on the enamel firing technique has been reported, so the aforementioned experimental results cannot be evaluated by comparison with those obtained using other methods. Instead, they can only be analyzed according to the experiments and relevant data in the current research. Analysis of the above experimental results shows that the mass of enamel specimens (decorations) has a real linear regression relationship with the firing duration. The linear regression equation was further applied and validated, with the obtained enamel specimens (decorations) all reaching the ideal effect. However, the following preconditions need to be met to ensure the representativeness and universal adaptability of the linear regression equation: enamel firing has to be performed according to “Experimental procedures” described in Section 1.2.2. For example, the regression equation established in the research was used in “Result application” in Section 2.2 to conduct enamel firing experiments on three different types of enamel specimens; three duplicates were prepared for each type of specimen for verification of enamel firing quality, and the resulting enamel specimens all reach the ideal effect (Figs 10 and 11). This proves the representativeness and applicability of the linear regression equation. If craftsmen use different temperatures or different models of muffle stoves for enamel firing, the proposed model (regression equation) cannot be used to calculate the firing durations. A certain number of enamel specimens need to be prepared at first to conduct enamel firing experiments, followed by establishment of a model (regression equation) according to the experiments, which can be utilized to guide subsequent enamel firing. This suggests that no matter what model of muffle stoves is used, craftsmen only need to set one temperature index for enamel firing when making enamel decorations. A quantitative enamel firing experiment can be designed in advance to establish the mathematical model (regression equation). Then, the model can be utilized to calculate the firing durations of each enamel specimen (decoration), thus achieving the goal of quantitative enamel firing as long as the specimens are fired according to the calculated firing duration.

## 4 Conclusion

Since being introduced to China in the Yuan dynasty, the art of enamel has been improved in the Ming dynasty, developed in the Qing dynasty, innovated and applied in modern times. The filigree enamel brand Cloisonné famous in China and abroad was founded in the Ming dynasty. Enamel-decorated furniture in the Qing dynasty has become one of the main characteristics of palace furniture of the Qing dynasty and is classed as a national treasure and precious cultural relic. The application field of enamels in the modern times has been further expanded, from enameled jewelry to enamel furniture and to interior and exterior decorations. Enamel craftsmanship is an intangible cultural heritage of China, of which enamel firing is a key link and the most important process, however, craftsmen of past dynasties all depended on their experience to fire enamels, that is, relying on their experience in heating duration and degree, so it is difficult to master the enamel firing technology and control the firing quality. The research explored the quantitative firing technology of enamels on copper pads. Through linear regression analysis, the mass of enamel specimens (decorations) has a real linear regression relationship with the firing duration. The linear regression equation constructed was used to calculate firing durations of different enamel specimens (decorations), which has been verified by enamel firing experiments. Therefore, the results provide a new idea and method and blaze a new path for realizing quantitative and standardized enamel firing. They positively affect the inheritance and innovation of traditional enamel craftsmanship and the improvement and development of the art of enamel to future benefit.

## Supporting information

Supporting Information files.docx (DOCX)
