# Supplementary material for: The quantitative enamel firing technique based on regression analysis
Source: PLoS One. 2025 May 19;20(5):e0322459. doi: 10.1371/journal.pone.0322459 (PMC12088522; doi:10.1371/journal.pone.0322459)

**The quantitative enamel firing technique based**

**on regression analysis**

**（Data file）**

**1 Experimental data processing**

Table 1 Experimental treatment levels

| Specimen | Specimen 1 (*X*_1_) | Specimen 2 (*X*_2_) | Specimen 3 (*X*_3_) | Specimen 4 (*X*_4_) | Specimen 5 (*X*_5_) | Specimen 6 (*X*_6_) |
| --- | --- | --- | --- | --- | --- | --- |
| Volume of copper pads | 50 × 40 × 2 mm  = 4000 mm^3^ | 80 × 50 × 2 mm  = 8000 mm^3^ | 120 × 50 × 2 mm = 12,000 mm^3^ | 100 × 80 × 2 mm = 16,000 mm^3^ | 10 0× 100 × 2 mm  = 20,000 mm^3^ | 120 × 100 × 2 mm  = 24,000 mm^3^ |
| Mass of copper pads | 35.6 g | 35.6 g × 2  = 71.2 g | 35.6 g × 3  = 106.8 g | 35.6 g × 4  = 142.4 g | 35.6 g × 5  = 178 g | 35.6 g × 6  = 213.6 g |
| Mass of enamel glazes | 2 g | 2 g × 2 = 4 g | 2 g × 3 = 6 g | 2 g × 4 = 8 g | 2 g × 5 = 10 g | 2 g × 6= 12 g |
| Specimen mass | 37.6 g | 37.6 g × 2  = 75.2 g | 37.6 g × 3  = 112.8 g | 37.6 g × 4  = 150.4 g | 37.6 g × 5  = 188 g | 37.6 g × 6  = 225.6 g |

Note: Copper pads were laser-cut and therefore had standard dimensions; through tests and calibration, the density of red copper was 8.9 g/cm^3^ in the calculation; the specimen mass = mass of copper pads + mass of enamel glazes.

**2 Enamel firing experiments test result data**

Table 2 Test results pertaining to specimens 1 after different firing durations

| Firing duration | Enamel firing effect |
| --- | --- |
| 100 s | The enamel glazes are not molten, which means that the firing duration is far from enough. |
| 110 s | The enamel glaze is not molten, which means that the firing duration is far from enough. |
| 120 s | The enamel glaze is not molten, which means that the firing duration is far from enough. |
| 130 s | Some enamel glazes begin to be molten, which means that the firing duration is far from enough. |
| 140 s | Some enamel glazes begin to be molten, which means that the firing duration is far from enough. |
| 150 s | Some enamel glazes begin to be molten, which means that the firing duration is far from enough. |
| 160 s | Some enamel glazes begin to be molten, which means that the firing duration is far from enough. |
| 170 s | Most enamel glaze has been molten, which means that the firing duration is obviously not long enough. |
| 180 s | The vast majority of enamel glaze has been molten, which means that the firing duration is still not long enough. |
| 190 s | The enamel glaze has been completely molten and the enamel shows ideal quality. |
| 200 s | The enamel glaze has been completely molten and begins to flow, which indicates that the firing duration is too long. |

Table 3 Test results pertaining to specimens 2 after different firing durations

| Firing duration | Enamel firing effect |
| --- | --- |
| 190 s | Most enamel glaze has been molten, which means that the firing duration is obviously not long enough. |
| 200 s | The vast majority of enamel glaze has been molten, which means that the firing duration is still not long enough. |
| 210 s | The enamel glaze has been completely molten and the enamel shows ideal quality. |
| 220 s | The enamel glaze has been completely molten while flows, which means that the firing duration is too long. |
| 230 s | The enamel glaze has been completely molten and begins to flow, which means that the firing duration is too long. |

Table 4 Test results pertaining to specimens 3 after different firing durations

| Firing duration | Enamel firing effect |
| --- | --- |
| 210 s | A small amount of enamel glaze is molten, which means that the firing duration is far from enough. |
| 220 s | Most enamel glaze has been molten, which means that the firing duration is obviously not long enough. |
| 230 s | The vast majority of enamel glaze has been molten, which means that the firing duration is still not long enough. |
| 240 s | The enamel glaze has been completely molten and the enamel shows ideal quality. |
| 250 s | The enamel glaze has been completely molten while flowed, which means that the firing duration is too long. |

Table 5 Test results pertaining to specimens 4 after different firing durations

| Firing duration | Enamel firing effect |
| --- | --- |
| 240 s | A small amount of enamel glaze is molten, which means that the firing duration is far from enough. |
| 250 s | Some enamel glazes have been molten, which means that the firing duration is obviously not long enough. |
| 260 s | Most enamel glaze has been molten, which means that the firing duration is obviously not long enough. |
| 270 s | The vast majority of enamel glaze has been completely molten, which means that the firing duration is still not long enough. |
| 280 s | The enamel glaze has been completely molten and the enamel shows ideal quality. |

Table 6 Test results pertaining to specimens 5 after different firing durations

| Firing duration | Enamel firing effect |
| --- | --- |
| 280 s | Most enamel glaze has been molten, which means that the firing duration is obviously not long enough. |
| 290 s | The vast majority of enamel glaze has been completely molten, which means that the firing duration is still not long enough. |
| 300 s | The enamel glaze has been completely molten and the enamel shows ideal quality. |
| 310 s | The enamel glaze has been completely molten while flows, which means that the firing duration is too long. |
| 320 s | The enamel glaze has been completely molten and begins to flow, which means that the firing duration is too long. |

Table 7 Test results pertaining to specimens 6 after different firing durations

| Firing duration | Enamel firing effect |
| --- | --- |
| 300 s | Some enamel glazes have been molten, which means that the firing duration is obviously not long enough. |
| 310 s | Most enamel glaze has been molten, which means that the firing duration is evidently not long enough. |
| 320 s | The vast majority of enamel glaze has been completely molten, which means that the firing duration is still not long enough. |
| 330 s | The enamel glaze has been completely molten and the enamel shows ideal quality. |
| 340 s | The enamel glaze has been completely molten and has flows, which means that the firing duration is too long. |

**3. Use SPSS 26.0 statistical software to analyze the relevant data obtained from the experimental results**


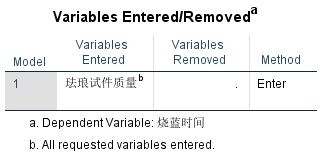


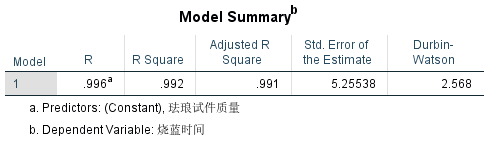


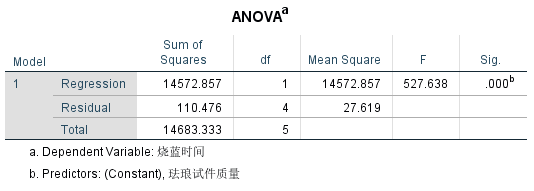


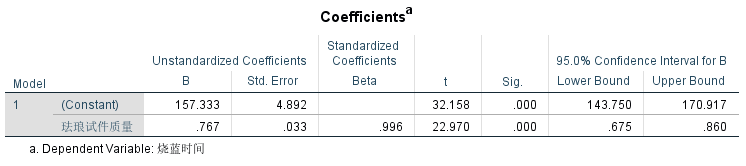


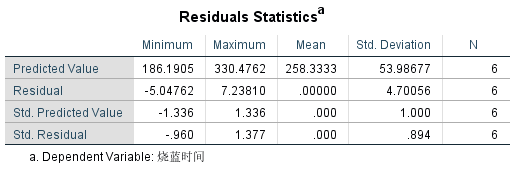


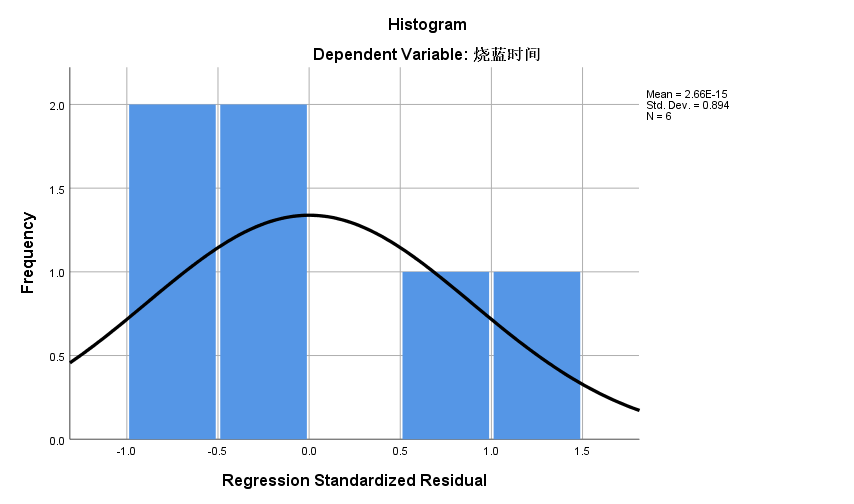


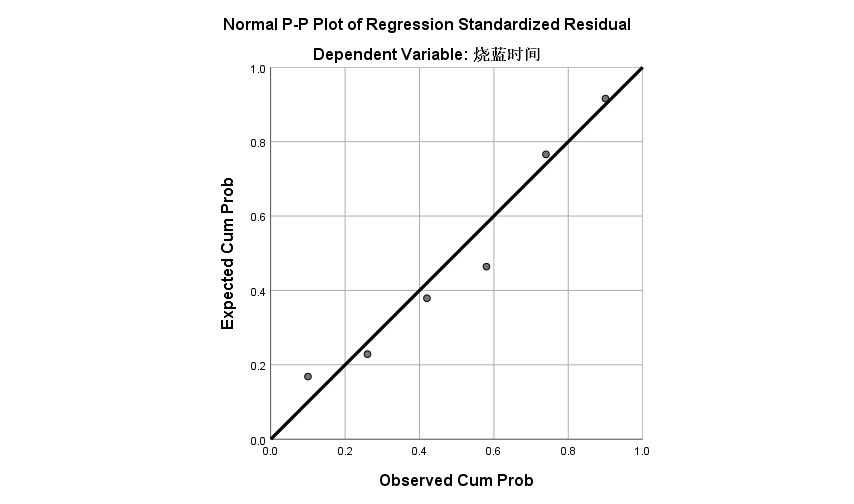

Supplement: Supporting Information files.docx — (DOCX) [file pone.0322459.s001.docx]
